# Automated analyser for monitoring the contents of hydrocarbons in gas emitted from exploratory bore-holes in the gas and oil industry

**DOI:** 10.1155/S1463924603000245

**Published:** 2003

**Authors:** Wacław Janicki, Wojciech Chrzanowski, Paweł Żwan, Jacek Namieśnik

**Affiliations:** 1 Department of Analytical Chemistry Chemical Faculty Gdańsk University of Technology 11/12 Narutowicza Street Gdańsk PL-80-952 Poland; 2 Department of Physical Chemistry Chemical Faculty Gdańsk University of Technology 11/12 Narutowicza Street Gdańsk PL-80-952 Poland; 3 Faculty of Electronics Telecommunication and Informatics Gdańsk University of Technology 11/12 Narutowicza Street Gdańsk PL-80-952 Poland

## Abstract

An automated analyser for total hydrocarbon contents and hydrocarbon composition (from methane to pentanes) was constructed and tested in both laboratory and field exploitation. It used two-channel analysis: continuous measurements of total hydrocarbon contents and periodic (90 or 150 s) composition analysis after separation of hydrocarbons on a gas chromatographic column. Flame ionization detectors were used in both channels. A simple 16-bit analogue-to-digital converter was used (4.8, practically four orders of magnitude), while the full measuring range (six orders of magnitude) was ensured by automatic dilution of the sample (or standard) with clean air. Full control of the operating (calibration/analyses) cycle was performed by microcomputer. An external programme, based on a computer provided with full information on the instrument operating conditions, presents the results of calibrations/analyses and enables them to be archived in a standard database used in the oil/gas drilling industry (N-LAB) by providing a suitable link. The instrument measuring range was 1 ppm to 100% with precision not worse than 5% at the detection limit. The analyser can operate autonomously for two months, recalibrating itself daily.


Journal of Automated Methods & Management in Chemistry
Vol. 25, No. 6 (November–December 2003) pp. 141–147

Taylor & Francis

Taylor & Francis Group

### Automated analyser for monitoring the contents of hydrocarbons in gas emitted from exploratory bore-holes in the gas and oil industry

**Wacław Janicki[1], Wojciech Chrzanowski[2]*, Paweł Żwan[3] and Jacek Namieśnik[1]**

*[1]Department of Analytical Chemistry, [2]Department of Physical Chemistry, Chemical Faculty, and [3]Faculty of Electronics, Telecommunication and Informatics, Gdańsk University of Technology, 11/12 Narutowicza Street, PL-80-952 Gdańsk, Poland*

*An automated analyser for total hydrocarbon contents and hydrocarbon composition (from methane to pentanes) was constructed and tested in both laboratory and field exploitation. It used two-channel analysis: continuous measurements of total hydrocarbon contents and periodic (90 or 150 s) composition analysis after separation of hydrocarbons on a gas chromatographic column. Flame ionization detectors were used in both channels. A simple 16-bit analogue-to-digital converter was used (4.8, practically four orders of magnitude), while the full measuring range (six orders of magnitude) was ensured by automatic dilution of the sample (or standard) with clean air. Full control of the operating (calibration/analyses) cycle was performed by microcomputer. An external programme, based on a computer provided with full information on the instrument operating conditions, presents the results of calibrations/analyses and enables them to be archived in a standard database used in the oil/gas drilling industry (N-LAB) by providing a suitable link. The instrument measuring range was 1 ppm to 100% with precision not worse than 5% at the detection limit. The analyser can operate autonomously for two months, recalibrating itself daily.*

### Introduction

Many branches of technology need chemical analyses, which usually do not impose serious problems for modern analytical equipment from a purely metrological point of view. The specificity of these analyses lies elsewhere. They usually must be performed in real time, continuously or with minimum delay between sampling and the results. Therefore, the instruments must be coupled on-line with the sample source, and sampling, sample preparation, its introduction into the analytical device, signal measurements, transformations and calculations leading to the final results must be performed automatically. Any calibration/recalibration steps involved should also be performed automatically. Additional requirements include robustness of the apparatus, serviceability by non-qualified personnel (not analysts or chemists), occasional portability, data storage for record keeping, and low cost. These features lead to the development of a subdiscipline of analytical chemistry, known as process analysis. Instruments employed in process analysis are usually not big, universal, expensive combines like those used in specialized analytical laboratories, but dedicated instruments, frequently customer tailored, and they are also known as 'fit-for-purpose' devices [1–3].

Our group was approached by a company supplying measuring equipment for field laboratories accompanying operations drilling exploratory bore-holes. Such a laboratory is usually housed in a container. Among measuring devices necessary for the drilling operation itself, a few analytical instruments are usually included. Composition of the gas emitted from the bore-hole, especially its hydrocarbon contents, is important from a geological point of view, may serve as an indicator when a gas- or oil-bearing layer is approached and is of importance from the occupational safety point of view. Several instruments are available on the market and used in the industry:

- Total gas detector and gas chromatograph (Petron Industries, Inc., Houston, TX, USA) [4].
- 8800 Series Hydrocarbon Analysers, Model 8550 Gas Analyser and 8900 Gas Chromatograph, Model 8550, Model 1015A Total Hydrocarbon Analyser (Baseline Industries, Baseline, Mocon, Lyons, CO, USA) [5].
- 3000 Micro GC (HP Micro GC), Agilent Technologies (formerly Hewlett-Packard Company Analytical Products Group; Palo Alto, CA, USA) [6].
- Gas chromatographs from 8600 Series, SRI Instruments (Torrance, CA, USA) [7].

Also, some general-purpose gas chromatographs, e.g. Tswett-500M, are used by the industry.

The specifications required include the following:

- Measuring range: 1 ppm to 100% total hydrocarbon contents (THC).
- Continuous THC measurements.
- Hydrocarbon composition (methane, ethane, propane, isobutane and *n*-butane, optionally isopentanes and *n*-pentane) analysed (at least) every 5 min.
- Full automation of analyses.
- Automatic calibration and daily recalibration.
- Autonomous, unattended operation period: two months.
- Results must be made available for an N-LAB database, which is used as a standard in the gas/oil industry.

The instrument thus designed and described in this paper was designated a model PPM automatic analyser of

* To whom correspondence should be addressed.
e-mail: wojtek@chem.pg.gda.pl

THC and light hydrocarbon composition in gases, especially from exploratory bore-holes.

### Operating principle

It was obvious that the instrument would have to include a two-channel design. In this design, a stream of gaseous sample is split and part of it directed to the THC analyser (continuous), while the other part is introduced periodically into a chromatographic column, as from the very beginning it was decided that gas chromatography will be employed for analysis of light hydrocarbon composition. The next logical conclusion was to employ the same type of detector in both channels. Flame ionization detector (FID) was chosen, as it can easily cover the required dynamic range. The real problem was transforming its signal into digital form. A 32-bit analogue-to-digital converter (ADC) is very expensive and requires sophisticated circuitry. Hence, to lower costs and avoid some deviations from linearity observed in the FID signal at very high concentrations of hydrocarbons (approaching 100%), it was decided to use a system permitting sample dilution before the analysis. Such a system had to be developed to perform automatic multipoint calibration of the THC channel. This permitted us to employ a simple, cheap, 16-bit ADC.

For the first test model, we envisaged a system where the dilution factor was changed on the basis of THC level alone. It worked well if no significant changes in hydrocarbon composition occurred. Note that the same concentrations (ppm vol.) of methane and propane produce FID signals differing by a factor of 3 (in favour of propane), while after gas chromatographic (GC) separation, a higher peak will be observed for methane, which is eluted at a very short retention time. These tests led to the development of a unit with two independent mixing chambers, each performing dilution (if required) for its own channel, increasing dilution when the signal exceeded 80% of the current measuring subrange (and ADC dynamic range). In the case of GC, it is based on the maximum of the highest peak (no matter which hydrocarbon it represents). Lowering dilution occurs when the defined signals fall below 10% of the subrange. The system of dilution factors and concentration subranges is shown in table 1. Note that unless concentrations in the real sample are not extreme, the detectors operate all the time at a similar concentration of hydrocarbons entering the detector. Assuming the gaseous standard mixture used for calibration (provided in a typical gas cylinder) is about 1% (vol.), the system permits a three-point calibration at concentrations 1430, 263 and 40 vppm (figure 6).

The GC module must operate in two modes: standard, covering C1–C4 hydrocarbons, and extended (optional) including C5. Automatic dosage of a sample using a six-port valve would be no problem unless higher hydrocarbons were present in the sample. Even if not eluted within the specified run time, they would stay in the column (some even accumulate), being eluted in successive runs, resulting in an unstable baseline and the occurrence of ghost peaks, which complicate interpretation of chromatograms. Note that in the standard mode, even the pentanes should be prevented from entering the GC column. This fact forced us to use a precolumn where higher hydrocarbons (5+ or 6+) are retained, while the lower ones enter the analytical column. During the analytical run, when the desired hydrocarbons are already being separated in the analytical column, the precolumn is backflushed, i.e. the carrier gas flow is reversed, thus removing the heavier hydrocarbons to the dump. Such an arrangement required use of a ten-port valve instead of a six-port one.

*Table 1. Dilution factors used in the THC and GC channels of the analyser and corresponding measuring subranges. A dilution factor equal to one means there is no dilution. Note that for concentrations above 0.017% (170 ppm), the THC detector actually operates within 0.017–0.4%.*

| Concentration in the real sample (%) | Dilution factor | Concentration in the analysed sample (%) |
|---|---|---|
| THC | | |
| 12–100 | 251 | 0.048–0.4 |
| 1.2–12 | 38 | 0.032–0.32 |
| 0.12–1.2 | 7.0 | 0.017–0.17 |
| < 0.12 | 1 | 0.0001–0.12 |
| GC | | |
| 5–100 | 500 | 0.010–0.2 |
| 0.25–5 | 50 | 0.005–0.1 |
| < 0.25 | 1 | 0.0001–0.25 |

Final parameters of the chromatographic system are as follows:

- Analytical column: 95 cm, SS, 1/8 inch, 2.1 mm i.d., packed, 20% squalane on Chromosorb P, 80–100 mesh.
- Precolumn: 15 cm, the same parameters and packing.

This system permits separation of C1–C4 hydrocarbons in 70 s and C1–C5 in 150 s, operated isothermally at 60°C and 20 $cm^3 min^{-1}$ carrier gas (hydrogen) flow rate (figure 4). Even shorter analysis times could be achieved (70 s in normal mode, 120 s in the extended mode; isothermally, temperature programming was out of the question for the sake of simplicity), but at the cost of methane/ethane resolution and possible overlapping of their two respective retention time windows. The optimum sample size (injection) was 200 μl.

Two versions of the instrument were constructed and tested, both in the laboratory and in the field. The design discussed below is the second one, which included changes resulting from observations made during exploitation of the earlier method. These permitted us to simplify the design, making the whole system more reliable. Among the changes was, for example, elimination of Tedlar® bags, which had served as an intermediate chamber for standard mixtures delivered from pressurized steel cylinders.

The design of the pneumatic system is shown in figure 1 and the actual arrangement of principal components

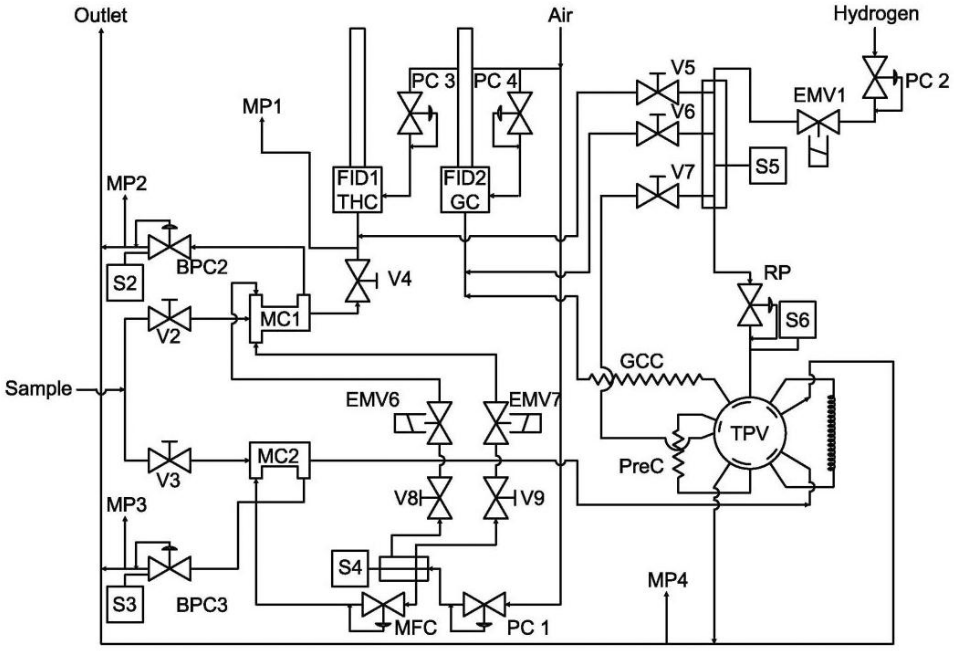

*Figure 1. Pneumatic system of a model PPM automatic analyser of the hydrocarbon contents in gas emitted from exploratory bore-holes: TPV, 10-port valve (shown in the 'load' position); GCC, gas chromatographic column; PreC, precolumn; MC1, THC sample/standard mixing chamber; MC2, GC sample/standard mixing chamber; MFC, mass flow controller; BPCx, back-pressure controllers; FID1, FID2, flame ionization detectors for THC and GC, respectively; Vx, needle valves for flow adjustment; EMVx, electromagnetic shut-off valves; PCx, pressure controllers; Sx, pressure sensors; RP, flow controller; MPx, flow checkpoints. The spiral to the right of TPV is a 200-μl injection loop.*

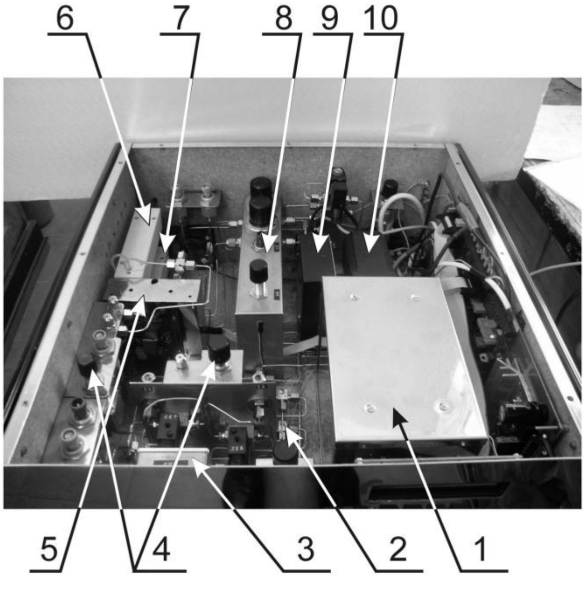

*Figure 2. Arrangement of the main units in the instrument housing: 1, thermostated chamber containing the GC column and precolumn; 2, MC2 (GC) mixing chamber, MC1 (THC) is visible behind MC2; 3, mass flow controller; 4, back-pressure controllers (GC, left; THC, right); 5, electrometer (shown with its cage open); 6, FID1 (THC); 7, FID2 (GC); 8, bank of pressure controllers; 9, 10-port valve controller; 10, 10-port valve motor operator.*

of the instrument is shown in figure 2. The instrument is housed in a single drawer (an earlier version used two drawers) and can be fitted in standard racks. Its weight is about 15 kg.

There are a few elements not shown in figure 1. Among the most important are: a pump (model TD-2LA; Brailsford & Co, Rye, NY, USA; 2 litres $min^{-1}$ at 0.07 MPa pressure), chosen for its robustness and a two-stage sample drier (freezing + permeation through a Nafion® tubing wall to the receiving hygroscopic agent—5A molecular sieve) [2]. Sample directly from the pump is split into two streams, with flows adjustable by means of needle valves V2 (THC line) and V3 (GC line). The mixing chamber MC1 permits one to add a suitable amount of dry, clean air to dilute the sample. The pressure at the outlet of the chamber is controlled by a back-pressure controller BPC2 to ensure the stability of the respective flow rates. Dry air is supplied at a constant pressure (adjusted by a pressure controller PC1). Dilution in MC1 is ensured by opening one of the two electromagnetic shut-off valves (EMV6 or EMV7), each providing a constant flow rate (adjusted initially by needle valves V8 and V9, respectively). If no dilution is needed, both EMVs are shut. This dilution method has been chosen because of the inertia of a mass flow controller that cannot be tolerated in the THC line, where the detector response is practically immediate. The accuracy of the dilution in this simplified system was quite satisfactory. A diluted sample from MC1 enters FID1 (THC line) via an extra needle valve V4.

A sample directed to the GC branch enters mixing chamber MC2 with a dry air stream provided by a mass flow controller MFC. The chamber outlet pressure is kept constant by means of another back-pressure controller (BPC3). The diluted sample enters the ten-port valve (TPV), which in figure 1 is shown in the 'load' position.

The sample flows through the dosing loop, filling it, and then to the dump. At the same time, hydrogen carrier gas flows through the analytical column (GCC), where separation occurs, and another stream of hydrogen back-flushes the precolumn (PreC). At the beginning of each run, TPV is switched to the 'inject' position, when sample from MC2 flows directly to the dump and the main hydrogen stream from the flow controller (RP) flows through the loop—collecting the sample—and the pre-column to the analytical column. TPV remains in the 'inject' position for 10 s in normal mode and for 15 s in the extended mode at the beginning of each run. Individual hydrocarbons eluted from GCC are directed to the FID2 detector. Both FIDs are supplied with suitable amounts of hydrogen and air for their burners.

Both columns and TPV are housed in a small thermostated chamber, ensuring a temperature of $50 \pm 1$°C. The needle valves (Vx) shown in figure 1 must be operated manually only when starting the instrument after its installation at a given site and, possibly, when some irregularities in its operation are observed. For this purpose, several flow checkpoints (MP) are provided, where the respective flows can be measured using a soap bubble flowmeter. Valves V8 and V9 are preset by the manufacturer and blocked to ensure the desired dilution in the THC line.

### Automation

Operation of the whole system is controlled by a preprogrammed microprocessor (digital microcontroller model W78E516B; Winbond, Electronics Corp., Hsinchu, Taiwan) on the basis of both time programme and simple logic decisions. The front panel of the instrument has a small liquid crystal display (LCD) and a 16-key keyboard permitting one to check the main operating parameters and to set those that do not need manual adjustment. This arrangement permits rapid checks and changes of parameter but requires memorizing a number of special codes (shortcuts). For this reason, the microcontroller is connected with an external computer by means of a typical serial interface (LPT, 19 200 baud rate, 8 data bits, 1 bit stop, no parity) and all the data are transmitted in a form of special packages—'frames' to the main programme. This is a two-way communication—frames received may contain instructions from the main programme to set chosen parameters. The microcontroller cannot interpret the signal, thus it also trasmits (during the run) the digitized signals to the computer.

The external programme (written in C++, Borland Builder v.5.0) operates under Windows (98 or newer, XP preferred) and may use 13 different windows. The structure of the programme tree is shown in figure 3. The programme may show captions and labels in Polish or English (instant switching is possible).

The main programme allows full control of the current state of the instrument, as well as: performing the calibration and storing calibration data, establishing the method of chromatogram interpretation (e.g. setting retention time windows), storing and recalling the methods, storing and recalling the results (chromatograms), signalling emergency situations (THC level

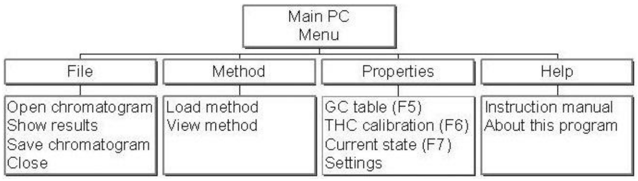

*Figure 3. Menu tree of the main control programme showing the main functions and operations permitted to be performed via the external computer.*

exceeding 1%), performing extensive self-diagnostic procedures and signalling possible errors or malfunctioning of the instrument. Finally, it provides a link to the N-LAB database used in the oil/gas industry for record keeping, where the final results are sent independently of their local 'on disk' storage.

Figure 4 shows the programme window (a chromatogram obtained earlier, which was loaded from the disk). A peak of methane, visibly present in the chromatogram, was not detected and integrated. It means that the method used (including retention time windows) was outdated and manual corrections should be introduced or the programme should be allowed to perform setting new time windows automatically. Usually automatic selection is correct; however, it is recommended to check the first chromatogram (at calibration) and to accept the method. Identification of peaks in the 'auto' mode is based on absolute retention time (ART) for methane and relative (versus methane) retention time (RRT) for the other peaks. If methane is absent in the sample (a rare case), ART is used for all peaks. Figure 4 shows the results when the instrument operates at the standard mode (a run ends after 90 s); pentanes are not detected.

Calibration is the most important step in operating the instrument, as it determines the accuracy of operation for a long time. Figures 5–7 present the three windows associated with this procedure. The calibration protocol is shown in figure 5. It includes concentrations of the respective hydrocarbons in the standard mixtures used (they can be set, if a new mixture is provided, in a separate window), date and time of calibration. The THC standard mixture contains only methane, therefore THC results are THC 'expressed as methane'. Therefore, they are usually higher than true content, and can even exceed 100% (100% propane, for example, would result in a 300% readout). We have developed a way to adjust the results by introducing a multiplication factor based on the last composition (chromatogram) available, but the industry prefers it this way (methane usually dominates the composition and at high concentrations—approaching and exceeding 1%—early warning is preferred). Figure 6 presents a three-point calibration curve obtained automatically (three consecutive dilutions applied, two 1-min lasting measurements for each dilution). Mean signals (arbitrary units, ADC output) for each dilution are displayed along with the regression parameters. Before feeding the THC with the diluted THC standard gas mixture, a 'zero' level (baseline) is established and reset using clean air. Figure 7 presents a GC calibration table (extended mode, pentanes included). Calibration factors are expressed as

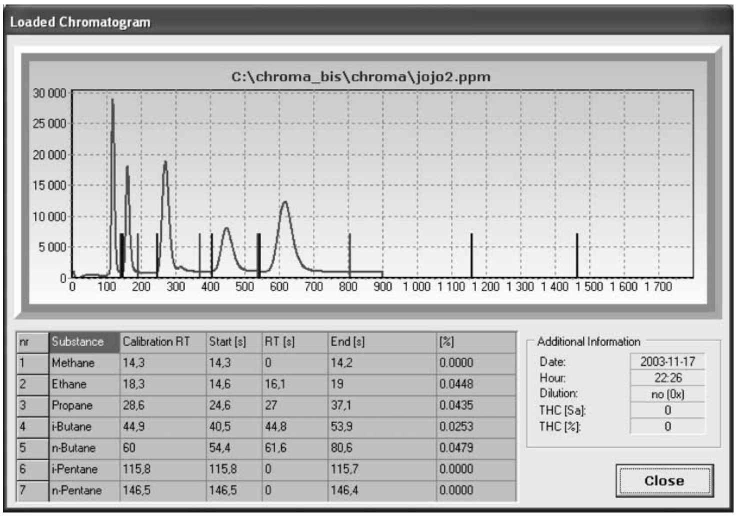

*Figure 4. Main programme window presenting a chromatogram and full GC analysis report of an earlier analysis—loaded manually from a file (time scale 100 units = 10 s).*

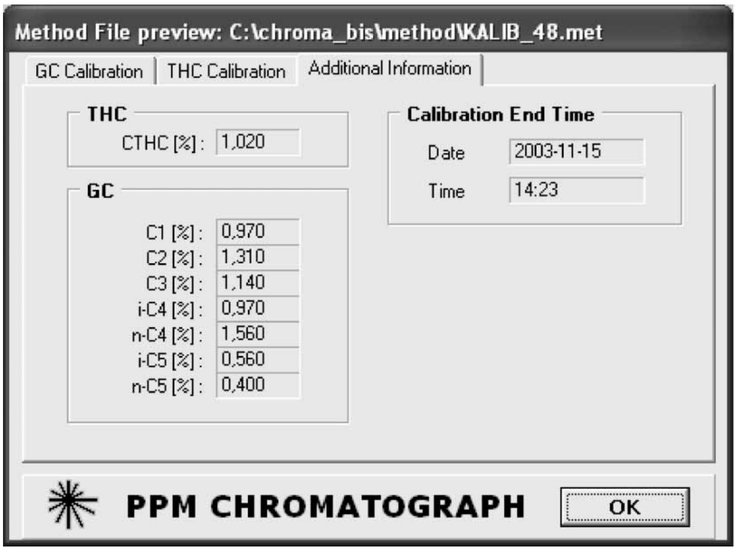

*Figure 5. Main programme window presenting a calibration time and composition of the standard mixtures used.*

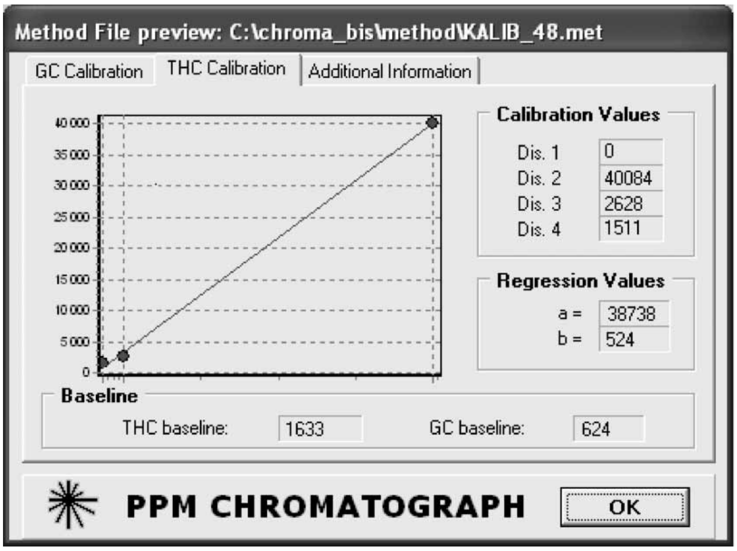

*Figure 6. Main programme window presenting an automatically taken THC three-point calibration curve.*

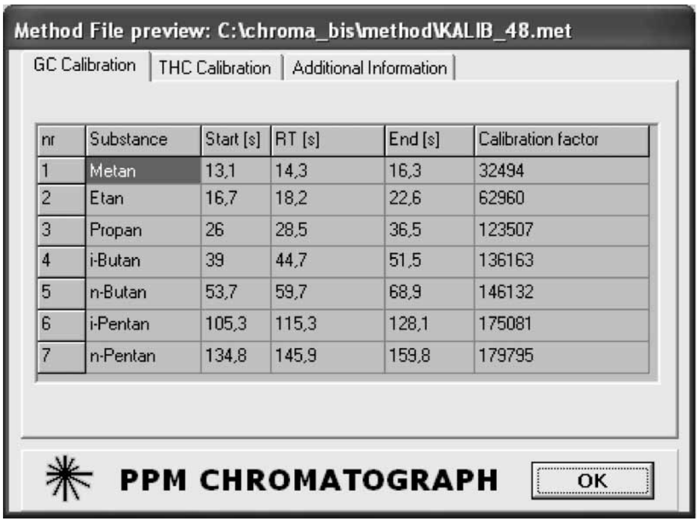

| nr | Substance | Start [s] | RT [s] | End [s] | Calibration factor |
|---|---|---|---|---|---|
| 1 | Metan | 13,1 | 14,3 | 16,3 | 32494 |
| 2 | Etan | 16,7 | 18,2 | 22,6 | 62960 |
| 3 | Propan | 26 | 28,5 | 36,5 | 123507 |
| 4 | i-Butan | 39 | 44,7 | 51,5 | 136163 |
| 5 | n-Butan | 53,7 | 59,7 | 68,9 | 146132 |
| 6 | i-Pentan | 105,3 | 115,3 | 128,1 | 175081 |
| 7 | n-Pentan | 134,8 | 145,9 | 159,8 | 179795 |

*Figure 7. Main programme window presenting GC calibration results. Calibration factors are AREA/AMT.*

AREA/AMT. Three consecutive GC runs with the same standard mixture are performed for complete calibration.

A recalibration procedure (for both THC and GC channels) is performed automatically every day at a time chosen when starting the instrument. Because the whole procedure takes about 30 min, it can be delayed if the measurements should not be interrupted for some reason, e.g. safety considerations when the THC concentration is high or if interesting developments in the bore-hole are expected.

### Results and conclusions from the laboratory and field tests

Laboratory tests included testing the performance of the modules like mixing–diluting systems, the extensive examination of both programmes (the microcontroller's and the main one), an automatic self-diagnostic system (most faulty situations had to be 'artificially created') and running the instrument with two sets of standards (one for calibration, another serving as a 'real' sample) for one month. After satisfactory conclusion of these tests, the instrument underwent a full two months of test exploitation under normal field conditions. During this period, it was operated by the rig team using the instruction manual and main programme help file. Assistance was provided at the first installation stage. We have thus obtained the first instrument ever manufactured for extensive check, repair and tests after a full year of operation at several sites in the Kazakhstan oil fields.

The principal conclusions resulting from these tests are as follows:

- Flow rates, once set at the first installation, are very stable and daily recalibration is almost unnecessary (hence, it was skipped frequently). Even the starting operation of the instrument after a few days' break without new calibration (using old calibration data) yielded results within 2–4% of the expected value (when a standard mixture was fed into the instrument).
- No deterioration of the GC system was observed over the guaranteed period of two-month operation (no significant baseline drift, ghost peaks or retention time shifts; retention times of the 'last' peaks especially had not become longer than the predicted run times). The Nafion drier molecular sieve load also appeared to last sufficiently long, even under field conditions.
- Number of errors indicated by the automatic self-diagnostic system could be reduced from the initial 19 to 12 (this was also possible due to elimination of the Tedlar bags).
- Real problem may be the quality of gases supplied to feed the instrument ('clean, dry' air for dilution, hydrogen carrier gas).
- Main features desired in the field appeared to be robustness and foolproofness due to frequent site changes and the quality of the personnel involved. The latter seems to be ensured by the self-diagnostic features included in the main programme. The former will be cared for; some improvements have already been made.

### Acknowledgements

The Department of Analytical Chemistry constitutes a 'Centre of Excellence in Environmental Analysis and Monitoring', which is a research project supported by the European Commission under the Fifth Framework Programme and contributing to the implementation of the Key Action 'Sustainable Management and Quality of Water' within the Energy, Environment and Sustainable Development (Contract No. EVK1-CT-2002–80010). The authors acknowledge this generous support and acknowledge the effective assistance of two electrical engineers: Cz. Gruszecki and M. Zaremski in designing the instrument control systems, Mr A. Wojdak in performing laboratory tests, and Mr Stanisław Żwan for performing field tests.

### References

1. Wasik, A., Lobinski, R. and Namieśnik, J., *Instrument Science and Technology*, **29** (2001), 393.

2. Janicki, W., Chrzanowski, W., Wasik, A., Przyk, E. and Namieśnik, J., *Journal of Automated Methods and Management in Chemistry*, **24** (2002), 9.
3. Krawczyk, M. and Namieśnik, J., *Journal of Automated Methods and Management in Chemistry*, **25** (2003), 115.
4. http://www.petronworld.com/prodserv/sensinst/chromato.htm and http://www.petronworld.com/prodserv/sensinst/totagas.htm (accessed 22 April 2004).
5. http://www.baselineindustries.com/product3.shtml and http://www.baselineindustries.com/product4.shtml (accessed 22 April 2004).
6. http://www.chem.agilent.com/Scripts/PDS.asp?lPage=1916 (accessed 22 April 2004).
7. http://www.srigc.com/2003catalog/cat-30.htm (accessed 22 April 2004).

